# Supplementary material for: Mapping the Americanization of English in space and time
Source: PLoS One. 2018 May 25;13(5):e0197741. doi: 10.1371/journal.pone.0197741 (PMC5969760; doi:10.1371/journal.pone.0197741)
Supplement: S1 File — Python code example. (PDF) [file pone.0197741.s001.pdf]

This file contains an example of a python code used to query the Twitter streaming API. The idea is to collect only geolocated tweets enclosed in a given geographical area BOX limited by latitudes y0 and y1, and longitudes x0 and x1, the two corners of BOX being (y0,x0) and (y1,x1). This code is only for illustrative purposes. Twitter developers may change the format of the queries at any moment. The updated information on how to access the Twitter API can be found in the user guide at https://developer.twitter.com/en/docs

```
from tweepy import Stream, OAuthHandler
from tweepy.streaming import StreamListener

CONSUMER_KEY = ''
CONSUMER_SECRET = ''
ACCESS_KEY = ''
ACCESS_SECRET = ''
BOX = [x0, y0, x1, y1]

class MyStreamListener(StreamListener):
	def on_status(self, status):
		print(status)

if __name__ == '__main__':
	auth = OAuthHandler(CONSUMER_KEY, CONSUMER_SECRET)
	auth.set_access_token(ACCESS_KEY, ACCESS_SECRET)

	listen = MyStreamListener()
	stream = Stream(auth, listen, gzip=True)
	stream.filter(locations=BOX)
```
