# Supplementary material for: Mapping the Americanization of English in space and time
Source: PLoS One. 2018 May 25;13(5):e0197741. doi: 10.1371/journal.pone.0197741 (PMC5969760; doi:10.1371/journal.pone.0197741)
Supplement: S2 File — Data source. (PDF) [file pone.0197741.s002.pdf]

The Google Books data information was downloaded from the American English and British English Ngram (N=1) viewer datasets available at https://storage.googleapis.com/books/ngrams/books/datasetsv2.html. These files enumerate how many times each word appears in the Google Books corpus each year.
